# A secreted Tapasin isoform impairs cytotoxic T lymphocyte recognition by disrupting exogenous MHC class I antigen presentation

**DOI:** 10.3389/fimmu.2024.1525136

**Published:** 2025-03-18

**Authors:** Robyn P. Seipp, Guillaume Hoeffel, Alexander R. Moise, Siri Lok, Anne-Claire Ripoche, Concepción Marañón, Anne Hosmalin, Wilfred A. Jefferies

**Affiliations:** ^1^ Department of Zoology, University of British Columbia, Vancouver, BC, Canada; ^2^ Michael Smith Laboratories, University of British Columbia, Vancouver, BC, Canada; ^3^ Université Paris Cité, CNRS, Inserm, Institut Cochin, Paris, France; ^4^ Vancouver Prostate Centre, Vancouver Coastal Health Research Institute, Vancouver, BC, Canada; ^5^ Centre for Blood Research, University of British Columbia, Vancouver, BC, Canada; ^6^ The Djavad Mowafaghian Centre for Brain Health, University of British Columbia, Vancouver, BC, Canada; ^7^ Department of Microbiology and Immunology, University of British Columbia, Vancouver, BC, Canada; ^8^ Department of Medical Genetics, University of British Columbia, Vancouver, BC, Canada; ^9^ Department of Urological Science, University of British Columbia, Vancouver, BC, Canada

**Keywords:** antigen presentation/processing, MHC class I, dendritic cells, cytotoxic T cells, Tapasin

## Abstract

Endogenous and exogenous antigen processing and presentation through the MHC class I peptide-loading complex (PLC) are essential for initiating cytotoxic T lymphocyte responses against pathogens and tumors. Tapasin, a key component of the PLC, is produced in multiple isoforms through alternative splicing, each isoform influencing the assembly and stability of MHC class I molecules differently. While the canonical Tapasin isoform plays a critical role in stabilizing MHC class I by facilitating optimal peptide loading in the endoplasmic reticulum (ER), the other isoforms function in distinct ways that impact immune regulation. This study aimed to investigate the role of Tapasin isoforms, particularly soluble isoform 3, in modulating antigen presentation and immune responses, focusing on their effects on MHC class I peptide loading and surface expression. Our findings show that isoforms 1 and 2 stabilize TAP and facilitate efficient peptide loading onto MHC class I in the ER, promoting optimal antigen presentation. In contrast, isoform 3, which lacks both the ER retention signal and the transmembrane domain, is secreted and acts as a negative regulator. Isoform 3 inhibits the loading of exogenous peptides onto MHC class I molecules at the cell surface, thereby playing a critical role in the spatial and temporal regulation of MHC class I antigen presentation. The secreted Tapasin isoform 3 likely regulates immune responses by preventing inappropriate T cell activation and cytotoxicity, which could otherwise lead to immune-mediated tissue damage and contribute to autoimmune disorders. Understanding the distinct functions of Tapasin isoforms provides insights into immune regulation and highlights the importance of fine-tuning peptide-loading processes to ensure proper immune responses and prevent immune-related pathologies.

## Introduction

The immune system relies on Major Histocompatibility Complex (MHC) class I and class II molecules to present antigens to T cells, enabling the body to detect and respond to pathogens and abnormal cells. MHC class I molecules present peptides derived from endogenous proteins, primarily to CD8+ cytotoxic T lymphocytes (CTLs), while MHC class II molecules present exogenous peptides to CD4+ helper T cells, playing a pivotal role in adaptive immunity (1). The process by which MHC class I molecules present antigens is tightly regulated and involves the peptide-loading complex (PLC), which includes proteins such as TAP (Transporter Associated with Antigen Processing), Tapasin, ERp57, calreticulin and the ER aminopeptidases (ERAP1/ERPA2) that play a pivotal role in the process (2). ERAAP, is the mouse analogue of ERAP1 in humans. These components ensure optimal selection and loading of peptides onto MHC class I molecules for effective CTL recognition (3).

Dendritic cells (DCs) are regarded as the most proficient professional antigen-presenting cells (APCs), capable of processing and presenting antigens on both MHC class I and class II molecules through an additional antigen processing and presentation process known as cross-presentation and cross-priming (4). This unique capacity allows DCs to present exogenous antigens on MHC class I molecules, thereby activating CD8+ T cells, which are essential for generating immune responses against viruses, tumors, and certain vaccines. Cross-presentation by dendritic cells (DCs) involves two primary pathways for processing exogenous antigens into peptides presented via MHC-I molecules to CD8+ T cells. In the cytosolic pathway, antigens are transported into the cytosol, degraded by the proteasome, and loaded onto MHC-I via the ER. In the vacuolar pathway, antigens remain in endosomes, where proteolysis occurs via cathepsins and direct MHC-I peptide loading occur. These mechanisms enable DCs to prime cytotoxic T cells against pathogens or tumors, even without direct infection of the DCs themselves (5, 6). Significant insights into these mechanism have emerged from studies demonstrating that CD74 and MHC class I cytoplasmic tail plays a crucial role in regulating the vacuolar pathway leading to DC cross-presentation and cross-priming (7). Modifications in this cytoplasmic domain can greatly affect the efficiency of antigen cross-presentation and the subsequent activation of CD8+ T cells (5, 8).

An intriguing aspect of antigen presentation involves trogocytosis, a process by which immune cells, including DCs, extract membrane fragments and associated molecules from other cells, facilitating antigen acquisition and immune synapse formation. We have shown that trogocytosis of MHC class I/peptide complexes from tumors and infected cells significantly enhances dendritic cell cross-presentation, leading to more effective activation of CD8+ T cells and promoting adaptive T cell responses (9–11). This finding provides valuable insights into how the immune system efficiently captures antigens from abnormal or infected cells, highlighting the importance of direct cell-to-cell interactions in immune surveillance.

Additionally, a pivotal study introduced the concept of “surrogate antigen processing,” revealing that antigenic peptides could be secreted in a TAP-dependent manner and subsequently reintroduced into cells for MHC class I presentation (12). This discovery expanded the understanding of antigen presentation, suggesting that extracellular peptides can be recaptured and presented by APCs, which has significant implications for immune responses against pathogens and tumor cells. This mechanism emphasizes the dynamic and adaptable nature of antigen processing and presentation pathways, offering further insights into how the immune system can utilize multiple routes to effectively display antigens for CD8^+^ T cell recognition.

Professional APCs, including DCs, macrophages, and B cells, are distinguished by their ability to efficiently process and present antigens while providing the requisite co-stimulatory signals for effective T cell activation (13). In contrast to DCs, non-professional APCs, such as fibroblasts and epithelial cells, have the ability to present antigens on MHC class I molecules but typically lack the necessary co-stimulatory molecules to activate naive T cells (14).

Tapasin is a crucial component of the PLC that enhances the loading of high-affinity peptides onto MHC class I molecules. This interaction is essential for maintaining a stable and immunogenic peptide repertoire on the cell surface (15). Studies have indicated that different protein isoforms of Tapasin may exert distinct roles in antigen presentation, particularly in stabilizing TAP and influencing the peptide repertoire presented to CTLs (16). Recent research has highlighted that Tapasin-mediated editing of the MHC class I immunopeptidome is epitope-specific and is dependent on peptide off-rate, abundance, and levels of Tapasin expression (17). Moreover, the dynamics of MHC class I proteins play a significant role in the Tapasin and TAP Binding Protein-Related (TAPBPR)-assisted editing processes, further refining the peptide repertoire available for T cell recognition (18).

Tapasin exists in multiple protein isoforms across vertebrate species, including humans, primates, rodents, and fish, with each isoform exhibiting distinct structural and functional properties that contribute to antigen processing and immune regulation (19–21). These isoforms are evolutionarily conserved, emphasizing their importance in immune mechanisms and potential therapeutic targets in diseases such as cancer and infections.

In humans, Tapasin isoforms, such as isoform 1, isoform 2, and isoform 3, arise from alternative splicing of mRNA and differ considerably in their amino acid sequences, influencing their interactions with MHC class I molecules and other PLC components (19, 22). Structural analyses utilizing advanced techniques like AlphaFold2 have provided insights into the structural distinctions among these isoforms, revealing critical regions that are conserved across species. For example, the N-terminal and immunoglobulin-like V domain, as well as the C-terminal immunoglobulin-like C domain, are highly conserved, indicating their essential functional roles in antigen processing (20). Additionally, AlphaFold2 has identified unique structural features in Tapasin, such as a conserved α-helix (residues A83 to T91) and a longer loop region (E72-G101) compared to TAPBPR, suggesting that these structural elements may play a role in Tapasin’s distinct mechanisms of interaction with MHC I molecules (20).

Although Tapasin isoforms are present across many species, comparative studies highlight both conserved and species-specific features. For instance, the overall architecture of Tapasin in humans is closely aligned with its homologs in species like the Sumatran orangutan (*Pongo abelii*), rat (*Rattus norvegicus*), mouse (*Mus musculus*), and zebrafish (*Danio rerio*) (20). While structural similarities indicate a conserved role in immune function, species-specific variations may affect the efficiency of antigen presentation and MHC class I assembly, reflecting evolutionary adaptations to different immune environments (17, 21).

In terms of functionality, the variability in Tapasin isoforms has important implications for MHC class I presentation. Studies show that higher expression levels of certain Tapasin isoforms enhance the loading of high-affinity peptides onto MHC class I molecules, thus improving immune recognition of pathogens (22, 23). For example, Isoforms 1 and 2 are primarily localized in the ER, where they stabilize the transporter associated with antigen processing (TAP), enhancing peptide loading and MHC class I surface expression (22). In contrast, Isoform 3, which lacks a transmembrane domain, is secreted from cells and negatively may regulate peptide loading, particularly for exogenous peptides. This divergence in function underscores the importance of understanding the specific roles of each isoform in immune regulation.

The evolutionary conservation of Tapasin and its isoforms highlights their critical role in immune responses across diverse species. The study of these isoforms, particularly through advanced structural and computational techniques, can provide valuable insights into the fundamental mechanisms of antigen presentation. Additionally, research into Tapasin’s functional diversity across species can inform the development of immunotherapies targeting immune evasion mechanisms in tumors and pathogens (24). Understanding the evolutionary dynamics and functional implications of Tapasin isoforms will likely uncover new strategies for enhancing immune responses in both clinical and therapeutic contexts.

In this context, recent studies suggest that certain isoforms of Tapasin may differentially regulate antigen presentation in non-professional APCs, potentially influencing immune responses against non-professional antigen sources. Understanding the molecular mechanisms of antigen presentation and the role of DCs in antigen processing and presentation is vital for developing therapeutic strategies, including vaccines and immunotherapies, aimed at enhancing the activation of CTLs against cancer and infectious diseases. This study aims to investigate the influence of Tapasin isoforms on antigen presentation and the role of dendritic cell cross-presentation in antigen capture, emphasizing their critical role in shaping the magnitude and quality of adaptive immune responses.

## Materials and methods

### Cell lines

Immortalized ear fibroblasts from C57Bl/6 mice, TAP1^-/-^ mice,. and Tapasin^-/-^ mice (25) (kind gifts from Dr. Luc Van Kaer (Vanderbilt University School of Medicine, Nashville, TN, USA), and the mouse dendritic cell line DC2.4 (26) were cultured in RPMI 1640 medium supplemented with 10% heat inactivated fetal bovine serum (HyClone, Logan, UT, USA), 2mM L-glutamine, penicillin (100 IU/ml), streptomycin (100 μg/ml), and 20mM HEPES.

### Cloning and construction of recombinant retroviruses encoding the Tapasin isoforms

**Figure 1:**
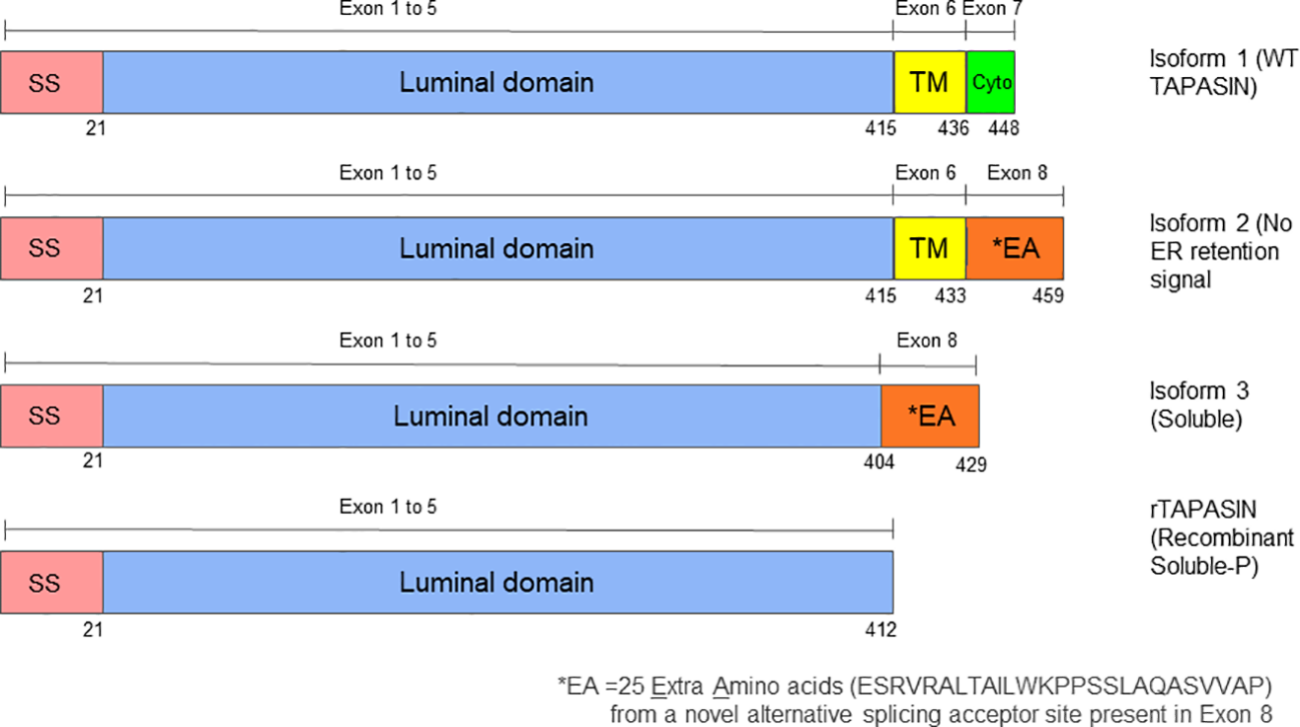
Structural comparison of Tapasin protein isoforms 1, 2, and 3. The diagram shows schematic representations of the three Tapasin isoforms studied. Tapasin has a total of eight exons. Isoform 1 represents the wild-type, full-length Tapasin protein, which includes a signal sequence, a luminal domain, a transmembrane domain, and a cytoplasmic domain with an ER retention signal. Isoform 2 contains a signal sequence, the entire luminal domain, most of the transmembrane domain, and the last three amino acids of the cytoplasmic domain but lacks the ER retention signal. Isoform 3 consists of a signal sequence, most of the luminal domain, and the last three amino acids of the cytoplasmic domain, lacking both the transmembrane domain and ER retention signal, resulting in a soluble form of Tapasin. Unlike the recombinant soluble Tapasin described by Wearsch and Cresswell (2011), this study investigates the naturally occurring Isoform 3 and the amino acid differences may explain the divergent functions of these soluble forms to Tapasin. SS refers to Signal Sequence, TM refers to Transmembrane domain and Cyto refers to Cytoplasmic domain.

Briefly, the Human full-length isoforms were subcloned into the pMXpie retroviral vector, and recombinant retroviruses encoding each isoform or vector alone were generated (**Figure 1**). These were used to stably transduce immortalized ear fibroblasts derived from Tapasin-/- mice (25) or the mouse DC cell line (with normal mouse Tapasin expression), DC2.4 (26). Transduced cells were selected in puromycin and normalized for transgene expression by sorting for equal GFP expression driven by IRES-GFP cassette encoded by the pMXpie retroviruses. Expression of the Human isoforms in these cells was confirmed at the RNA level by RT-PCR using primers capable of amplifying all three isoforms, as well as by Western blot or immunoprecipitation followed by Western blot for detection of expression of the isoforms at the protein level. Both RT-PCR and Western blot bands were of the expected size for each isoform.

### Antibodies

The rabbit antiserum against mouse and rat TAP1 protein was previously described (27). Rabbit anti-Human Tapasin antibodies (StressGen Biotechnologies Corp, Victoria, BC, Canada), rabbit anti- Mouse Tapasin antibodies gift from Dr. David Williams, University of Toronto, The rabbit serum against mouse and rat TAP2 (116/4) was kindly provided by Dr. Geoff Butcher (University of Cambridge, Cambridge, UK). Rabbit anti-Tapasin antiserum number 2668 was provided courtesy of Dr. Ted Hansen, Washington University School of Medicine, St. Louis, MO, USA) (28). Primary antibodies used were all used at 1/100 dilutions and were obtained from BDPharmingen (Mississauga, ON) except where indicated. These included PE anti-H-2K^b^ (clone AF.6-88.5) and PE anti-I-Ab (clone AF6-120.1). For H-2D^b^ staining, undiluted supernatant from the 28.14.8S hybridoma (164) was used with a PE anti-mouse IgG + IgM F(ab’)2 (H+L) secondary (Jackson Immunoresearch) diluted 1/200. For H- 2K^b^/SIINFEKL complex detection, biotinylated antibody from the 25.D1.16 hybridoma (165) was used at 1/100 dilution with PE Streptavidin secondary (Jackson Immunoresearch) at 1/200 dilution. For all flow cytometry involving DC2.4 cells, cells were first incubated with Fc blocker (anti-mouse Fcγ III/II receptor clone 2.4G2, BD Pharmingen) diluted 1/100 for 30 min prior to staining with specific antibodies.

### Flow cytometry

The effect of the Tapasin isoforms on MHC I surface expression levels was examined in Tapasin-/- fibroblasts expressing each isoform or pMX-pie vector alone by flow cytometry with conformation-dependent antibodies against the two MHC I alleles expressed by these cells: H-2K^b^ and H-2D^b^. Cells were incubated at 4°C with saturating amounts of PE anti-H-2K^b^ (clone AF.6-88.5) antibody (BD Pharmingen, Mississauga, ON) for 30 min, washed three times in PBS and analyzed on a FACSCalibur or FACScan (BD). Results were analyzed with FlowJo version 4.5.9. For H-2K^b^/SIINFEKL complex detection, biotinylated antibody from the 25.D1.16 hybridoma (29) was used with PE-Streptavidin secondary (Jackson Immunoresearch). Unless otherwise specified the secondary antibody alone was used as the negative control.

### Chromium-release assay

Vesicular Stomatitis Virus, Indiana Strain (VSV), a gift from Frank Tufaro (University of British Columbia, Vancouver, Canada), was cultured on Vero cells (ATCC). Cytotoxic T lymphocytes (CTL) were generated by intraperitoneal (i.p) inoculation of C57BL/6 mice with 3x10^6^ tissue culture infectious particles (TCIP) of VSV. Splenocytes were harvested seven days later, separated into single cells, washed three times with PBS and incubated for 5 days at 37°C, 5% CO_2_ in 20 mL “CTL media” (RPMI-1640 containing 10% heat inactivated FBS (Hyclone), 20 mM HEPES, 100 IU/ml penicillin, 100 μg/ml streptomycin, 0.1 mM non-essential amino acids, 1 mM Na-pyruvate, and 50 μM 2-mercaptoethanol) per spleen (approximately 10^6^cells/ml) with 1μM of VSV-NP52-59 immunodominant peptide (RGYVYQGL). The day of the assay, CTLs were harvested, washed three times, counted and mixed with target cells at the indicated ratios. For target cells, cells were infected for 6 hrs with VSV at a multiplicity of infection (MOI) of 200, or left uninfected as negative controls or pulsed with VSV-NP52-59 exogenous peptide as positive controls. Cells were washed, resuspended in 200ul of CTL media per 2x10^6^ cells and incubated with 10 μl (100 μCi) of ^51^Cr (as sodium chromate; Amersham Biosciences) for 1 hr. Cells were washed 3X in PBS, counted and mixed with effector CTLs at the indicated ratios for 4 hrs at 37°C, 5% CO2. For maximal/minimal counts, target cells were either lysed with 5% Triton X-100 or incubated with media lacking CTLs, respectively. Following the 4 hr incubation, cells were gently spun, 100 μl of supernatant was removed and quantified in a gamma counter (LKB Instruments, Gaithersburg, MD). To calculate percent specific killing, the following formula was used: ((experimental - minimum control)/(maximum – minimum control)) x 100%. Cytotoxicity was measured in a standard 4 hr ^51^Cr-release assay.

### Real time quantitative RT-PCR

Cells were washed twice in PBS and frozen in RNAlater (Ambion), then total RNA was isolated with Trizol (Invitrogen) and DNase-I digested to remove genomic DNA contaminants. Equal amounts of RNA per sample (600-800ng, depending on RNA yield for each repetition) were converted to cDNA with Superscript II and OligodT as per the manufacturer’s instructions, followed by RNase H digestion. qRT-PCR using a Roche Light Cycle and SYBR Green Taq ReadyMix (Sigma) was used to evaluate expression of the different isoforms, normalized with the amount of ribosomal S15 in each sample using standard curves for each primer set. A common forward primer within exon 5 (5’-CACCACTGGCAGCATGGGGCACGC-3’) was used for all three isoforms, while the isoform 1-specific reverse primer spanned exon 7 and 8 (5’-TCACTCTGCTTTCTTCTTTGAATCCTT G -3’), the isoform 2-specific primer spanned exon 6 and 8 (5’-TCACTCTGCTTTCAGCCC-3’), and the isoform 3-specific primer spanned exon 5 and 8 (5’-TCACTCTGCTTTCTGCTA -3’). These isoform-specific primers yielded PCR products of 235 bp, 200 bp, and 110 bp for isoforms 1, 2 and 3, respectively.

### ERp57 co-immunoprecipitation with Tapasin isoforms

Immunoprecipitation, co-immunoprecipitation and Western blots were performed on 400 μl of lysate made from 2.5x10^6^ Tapasin^-/-^ fibroblasts expressing Tapasin isoforms or vector alone, lysed as described in 1% NP-40 buffer. One hundred microliters were removed for Western blot for ERp57 from whole cell lysates. To immunoprecipitate human Tapasin, the remaining lysate was precleared for 2hrs rotating at 4°C with 50 μl of Protein G sepharose, and incubated overnight precipitated with 1 μl of PaSta.1 anti-human Tapasin mouse monoclonal (courtesy of Dr. Peter Cresswell, Yale University) and 50 μl of Protein G sepharose. Samples were washed 3X in 1% NP-40, mixed with 80 μl of 2X protein sample buffer, separated by SDS-PAGE in duplicate and probed by Western blot on PDVF membranes with either the Rgp48N rabbit polyclonal to human Tapasin or rabbit anti-ERp57 (Stressgen #SPA- 585). Both primary antibodies were used at 1/2000 dilutions with HRP conjugated antirabbit secondary antibody (Jackson ImmunoResearch) at 1/10,000.

### Metabolic labeling, pulse-chase and endoglycosidase H experiments

Tapasin^-/-^ cells expressing each Tapasin isoform were collected (5x10^6^ cells/sample), washed once in Cystine/Methionine-Free DMEM (CellGro Cat. No. 17- 204-CI, Mediatech, Herndon VA) + 5% FBS, then once in PBS. Cells were resuspended in Cys/Met-Free DMEM + 5% FBS and plated in 6-well plates (one well per time point) for one hour of starvation and adherence to the plates. Ten microliters (~20 μCi) of Pro-Mix L-^35^S *in vitro* Cell Labeling Mix (Amersham Biosciences) were added per well for 15 min. Cells were then placed on ice, washed once with ice-cold PBS and normal DMEM + 10% FBS and cold Cys/Met for chase. At given time points, samples were removed and placed on ice, washed once in PBS and lysed in 1mL of TX-100 lysis buffer for 30 min, spun at 15 min at 4°C 10,000Xg, and pre-cleared overnight with pre-washed Protein G sepharose beads (Amersham Biosciences) and normal rabbit serum. The amount of labeled protein in each sample was quantified by TCA precipitation and counted in a scintillation counter. Normalized amounts of each sample were precipitated with antibodies to either H-2K^b^ (P8, recognizing all conformations of H-2K^b^, courtesy of Jacques Neefjes, The Netherlands Cancer Institute) or human Tapasin (PaSta.1) for several hours or overnight, followed by binding to Protein G sepharose beads for one hour with rotation at 4°C. Samples were washed three times with 0.1% TX-100 in TBS and split into two separate samples: each was suspended in EndoH buffer but EndoHf enzyme (200 mIUB, New England Biolabs, P0703S) was added to only one sample. Both samples were incubated overnight at 37°C, then mixed with 2X protein sample buffer and separated by SDS-PAGE (12% separating/5% stacking). Gels were dried and exposed to a Phosphorimaging screen for 10 days, followed by visualization with a Phosphorimager SI (Molecular Dynamics), ImageQuant 5.2 software version 4.0, scanning at 100micron, PMT voltage 700.

### Thermostability of MHC class I molecules

In order to assess the stability of surface MHC I, *de novo* MHC I being synthesized and assembled in the ER must be blocked from reaching the cell surface and replenishing the MHC I being turned over. Brefeldin A (BFA) was used to block ER to Golgi transport of MHC, followed by measurement of surface H-2K^b^ by flow cytometry with a conformation-dependent antibody at various time points after BFA addition at 37°C. The control was MHC I expression on untreated cells.

### Exogenous SIINFEKL loading assay

Cells were harvested, counted, and approximately 5x10^6^ cells were incubated in their respective supernatants with indicated amounts of SIINFEKL for 1 hr at 37°C, 5% CO_2_, then washed three times in cold PBS, stained at 4°C for H-2K^b^ or H-2K^b^/SIINFEKL and analyzed using flow cytometry. In experiments involving Brefeldin A (BFA), cells were first incubated with 10 μg/ml of BFA for 30 min prior to addition of SIINFEKL to the BFA-containing media, then assayed as per above. To detect an effect from soluble isoform 3, a 150 mm dish of Tapasin^-/-^ fibroblasts were incubated overnight with 0.45 μm-filtered supernatant from a confluent dish containing Tapasin^-/-^ fibroblasts expressing isoform 3, and assayed as described with vector alone and isoform 3-expressing Tapasin^-/-^ fibroblasts as controls.

### Cross-presentation assays

Dendritic cells (DC2.4) were incubated overnight with indicated concentrations of soluble ovalbumin (Worthington Biochemical Corp., Lakewood, NJ) in PBS. The following morning, one well was pulsed with SIINFEKL peptide at 1 μg/ml for 1 hr at 37°C, and cells from all samples were collected, washed 3 times with PBS and fixed for 10 min with 0.005% glutaraldehyde. Cells were resuspended in complete RPMI and either stained for flow cytometry with antibodies against total H-2K^b^ or with the 25.D1.16 antibody specific for H-2K^b^/SIINFEKL complexes, or 1x10^5^ cells were mixed 1:1 with B3Z hybridoma T cells overnight in 96 well plates. The following morning, cells were lysed in 100 μl of CPRG (Chlorophenol red-B-D-galactopyranoside, Roche) solution (91mg CPRG + 1.25ml NP-40, 9ml 1M MgCl_2_ made up to 1 litre in PBS) and plates were read at 595 nm subtracting 655 nm background at 24 or 48 hrs to obtain a measure of the production of the β-galactosidase reporter under NF-AT elements by the B3Z TCR recognizing H-2K^b^/SIINFEKL complexes (30).

### Generation of recombinant retroviruses and infection of cell lines

Recombinant retroviruses encoding the human Tapasin isoforms were generated by co-transfection of pMXpie plasmid encoding the Tapasin isoform cDNA with pEco plasmid into BOSC23 cells plated in 10cm plates at 80% confluence using FuGene6 (Roche) according to manufacturer’s instructions. The supernatant containing recombinant retrovirus was collected and pooled over 4 days, filtered and flash frozen in aliquots.

Immortalized ear fibroblasts from Tapasin^-/-^ mice generated by serial passaging were infected with recombinant retroviruses, assessed for expression of GFP from the IRES-GFP cassette after 48hrs, and selected with 6 μg/ml puromycin for approximately two weeks until the majority of cells appeared GFP-positive. Cells expressing equal levels of GFP were sorted on a FACSVantage cell sorter from each group of transduced cells and maintained in DMEM media with 5 μg/ml puromycin thereafter, with additional resorting if GFP levels diverged between populations.

### Statistical analysis

Statistical analysis of qRT-PCR, flow cytometry and B3Z assays was performed with GraphPad Prism 4.00 software for Windows (GraphPad Software, San Diego CA, USA) using a multiple one-way ANOVA with Tukey’s post-test. Error bars represent the standard error of the mean (SEM).

## Results

### Effect of the Tapasin isoforms on MHC class I surface expression in fibroblasts from Tapasin^-/-^ mice

**Figure 2:**
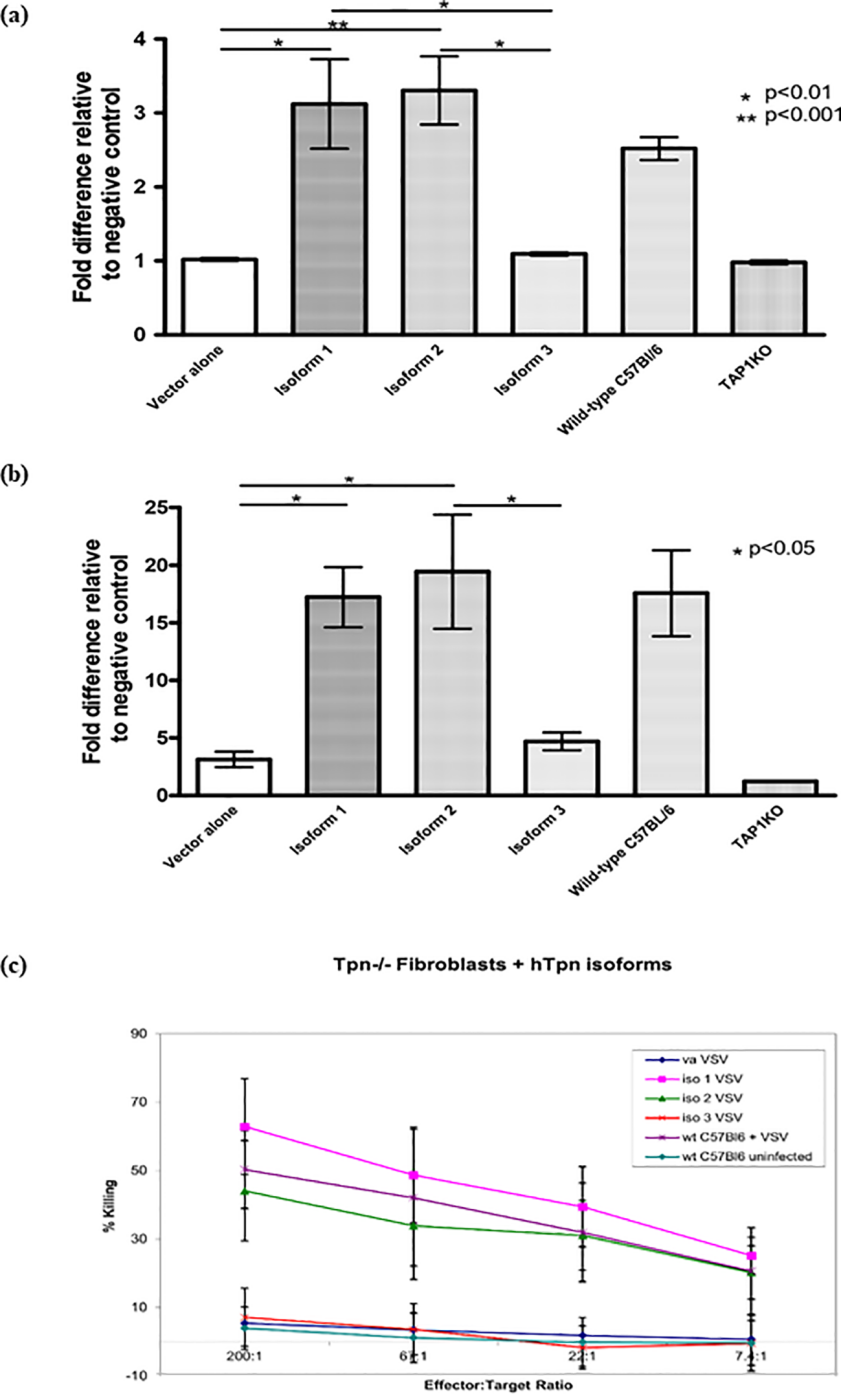
Tapasin Protein Isoform 1 and 2, but not Isoform 3, Complement Antigen Presentation and Restore CTL-Mediated Killing in Tapasin-Deficient Fibroblasts. Flow cytometry analysis showed that Isoforms 1 and 2 complemented the expression of both H-2K^b^ **(A)** and H-2D^b^ **(B)**, restoring surface MHC class I levels to wild-type levels in Tapasin^-/-^ fibroblasts. In contrast, Isoform 3 did not complement expression, resulting in very low surface MHC class I levels, comparable to vector control and TAP1-deficient fibroblasts. These findings highlight the functional differences between the isoforms in supporting MHC class I surface expression. Data represent results from three independent experiments. **(C)** ^51^Cr-release assay using VSV-infected Tapasin-deficient fibroblasts expressing different Tapasin isoforms as targets for VSV-specific CTLs was undertaken. Tapasin-deficient fibroblasts expressing Isoforms 1 and 2, when infected with VSV, were efficiently killed by VSV-specific CTLs, indicating successful antigen presentation. This demonstrates that expression of Isoforms 1 and 2, but not Isoform 3, complements the ability to present VSV antigens and elicit CTL-mediated killing. In contrast, cells expressing vector alone, Isoform 3, or uninfected Tapasin^-^control cells were not killed. Data shown are representative of one experiment, with similar results obtained in three independent repeats. Vector alone is designated as "va".

The effect of the Tapasin isoforms on MHC I surface expression levels was examined in Tapasin^-/-^ fibroblasts expressing each isoform or pMX-pie vector alone by flow cytometry with conformation-dependent antibodies against the two MHC I alleles expressed by these cells: H-2K^b^ and H-2D^b^. Tapasin^-/-^ fibroblasts naturally express very low surface MHC class I (25, 31), similar to TAP1^-/-^ fibroblasts (32). Expression of isoform 1 and 2 resulted in restoration of surface H-2K^b^ and H-2D^b^ expression to levels similar to C57BL/6 fibroblasts (**Figure 2**). However, isoform 3 expression was not able to restore the surface MHC I expression of either allele, and the low levels were statistically no different from those of Tapasin^-/-^ fibroblasts expressing vector alone or TAP1^-/-^ fibroblasts.

### Effect of the Tapasin isoforms on susceptibility to specific CTL killing of fibroblasts from Tapasin^-/-^ mice

Classical ^51^Cr release CTL assays were performed to determine whether the increase in surface MHC I expression in Tapasin^-/-^ fibroblasts mediated by isoform 1 and 2 resulted in restoration of the ability of the cells to be recognized and killed by specific CTLs. Tapasin^-/-^ fibroblasts expressing each isoform or vector alone were infected with Vesicular Stomatitis Virus (VSV), loaded with ^51^Cr, and incubated with splenic CTLs specific for a VSV immunodominant peptide (VSV-NP_52-59_) in the context of H-2K^b^, which were isolated from C57BL/6 mice infected with VSV. As positive and negative controls, C57BL/6 fibroblasts were infected with VSV or left uninfected, respectively. Tapasin^-/-^ fibroblasts expressing each isoform or vector alone that were not infected with VSV were also included as additional negative controls and showed virtually no susceptibility to killing (data not shown). Consistent with the flow cytometry results, Tapasin^-/-^ fibroblasts expressing isoform 1 and 2 were recognized and killed as efficiently as C57BL/6 fibroblasts, while Tapasin^-/-^ fibroblasts expressing vector alone or isoform 3 were not killed, similar to the uninfected C57BL/6 negative control. Killing efficiency decreased when decreasing numbers of CTLs were mixed with the target cells in a titration curve, indicating that the specific CTLs were mediating the cell lysis (**Figure 2C**).

### Ability of Tapasin isoforms to stabilize TAP

**Figure 3:**
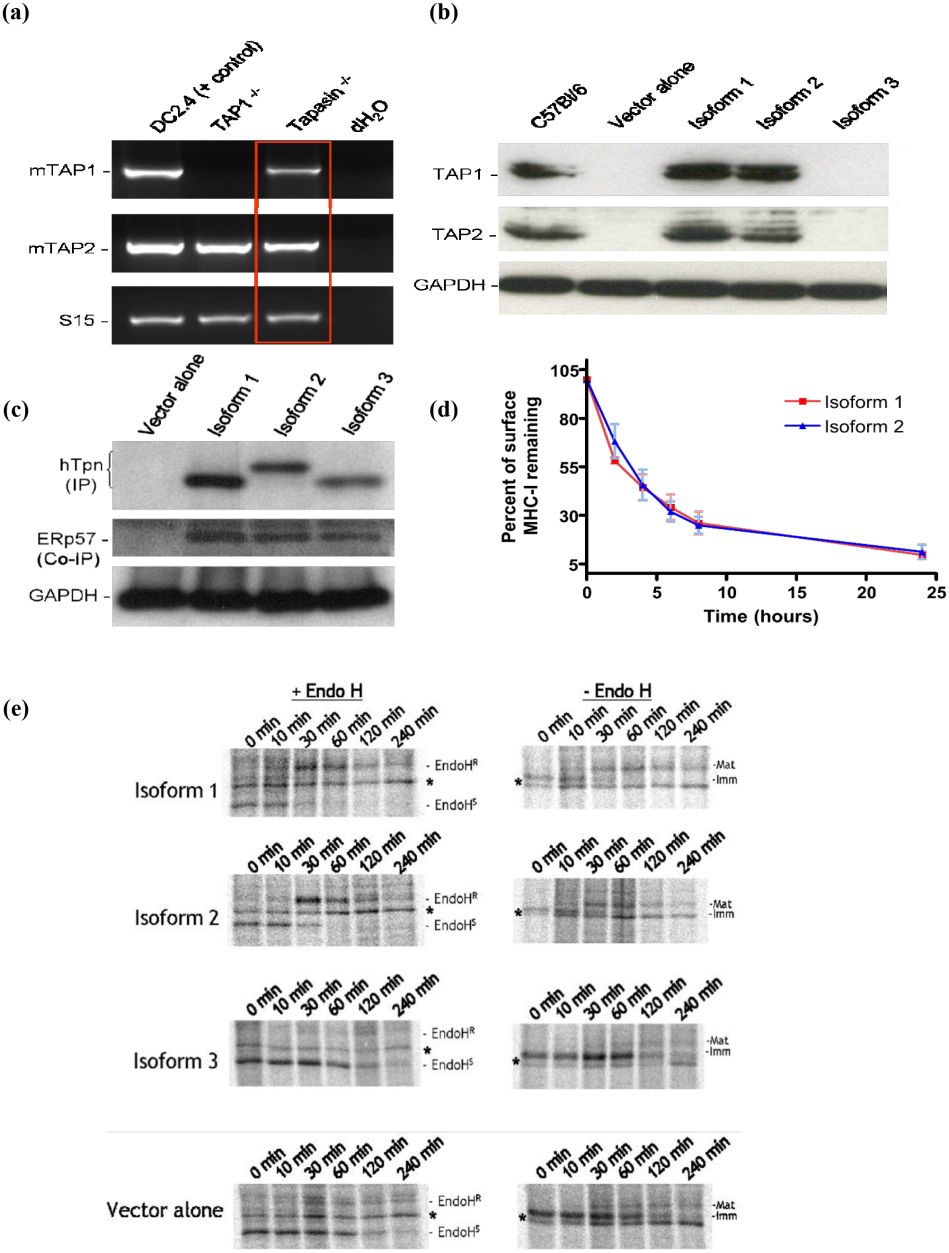
Tapasin isoforms 1 and 2 restore stabilization of TAP1 and TAP2 protein levels in Tapasin-/- fibroblasts but not Tapasin isoform 3. ERp57 interacts with all three Tapasin isoforms. Isoforms 1 and 2 Induce Similar Thermal Stability of MHC class I on the Surface of Tapasin-/- Fibroblasts. Unlike Isoform 3, Isoforms 1 and 2 Restore Proper Trafficking and Glycosylation of MHC class I in Tapasin-/- Fibroblasts **(A)** RT-PCR indicates that Tapasin^-/-^ fibroblasts express mouse TAP1 and TAP2 mRNA (DC2.4 DC cell line = positive control for mTAP1 and mTAP2 mRNA, TAP1^-/-^ = negative control for mTAP1 mRNA, S15 = mRNA loading control for RT-PCR). **(B)** TAP1 and TAP2 protein levels are undetectable by Western blot of Tapasin^-/-^ fibroblasts expressing vector alone or isoform 3, but are stabilized at levels similar to C57BL/6 wildtype fibroblasts upon expression of isoforms 1 and 2. GAPDH = protein loading control (~35kDa). Both TAP1 and TAP2 proteins are ~70kDa in size. Data shown represents the results of one experiment, which was repeated twice. **(C)** Tapasin^-/-^ fibroblasts expressing the Tapasin isoforms or vector alone were immunoprecipitated with an anti-human Tapasin monoclonal antibody and probed with a rabbit anti-human Tapasin polyclonal (top panel) or rabbit anti-mouse ERp57 polyclonal (middle panel). ERp57 co-immunoprecipitated with all three Tapasin isoforms. GAPDH = protein loading control from lysates used for immunoprecipitation (IP) or co-immunoprecipitation (Co-IP). **(D)** The thermal stability at 37°C of surface H-2K^b^ molecules in Tapasin^-/-^ fibroblasts expressing Tapasin isoforms 1 or 2 is very similar. Surface H-2K^b^ molecules, measured by flow cytometry, decay at similar rates upon blockage of *de novo* MHC I ER exit, suggesting the peptides loaded onto H-2K^b^ in these cells by the two isoforms are similar in their binding affinity and ability to stabilize this MHC I molecule. Data shown were compiled from three independent experiments. **(E)** Pulse-chase and EndoH digestion assay of H-2K^b^ maturation rate in Tapasin^-/-^ fibroblasts. Isoforms 1 and 2 allow H-2K^b^ molecules to traffic through the Golgi and acquire EndoH resistance and higher molecular weight glycosylation in Tapasin^-/-^ fibroblasts at similar rates, with acquisition of EndoH resistance - and a concurrent increase in molecular weight in undigested controls due to higher glycosylation - beginning at ten minutes and completed by sixty minutes. In contrast, very few H-2K^b^ molecules acquire EndoH resistance or higher glycosylation in cells expressing vector alone or isoform 3, indicating that the majority of H-2K^b^ in these cells remains in the ER. H-2K^b^ was immunoprecipitated with the P8 polyclonal antibody recognizing the C terminal region of H-2K^b^ in both folded and unfolded molecules. * Non-specific, unidentified band found in all samples. EndoH^R^ = Endoglycosidase H resistant, EndoH^S^ = Endoglycosidase H sensitive. Data shown represents the results of one experiment, which was repeated three times. Refer to **Supplementary Table 1** for densitometry data from pulse-chase experiments.

The underlying mechanism governing the ability of isoforms 1 and 2 to restore MHC I surface expression and present specific peptides to CTLs was revealed by Western blotting for mouse TAP1 and TAP2 protein levels in Tapasin^-/-^ fibroblasts expressing each isoform. Although mouse TAP1 and TAP2 mRNA was expressed normally in Tapasin^-/-^ fibroblasts (**Figure 3A**), TAP1 and TAP2 protein levels in these cells were undetectable (see “vector alone” lane, **Figure 3B**). Tapasin is known to stabilize TAP1 and TAP2 proteins, and it is the transmembrane domain of Tapasin that mediates its interactions with the TAP heterodimer (33–35). Isoforms 1 and 2 retain the Tapasin transmembrane domain and were therefore able to stabilize mTAP1 and TAP2 protein to levels similar to C57BL/6 cells (**Figure 3B**). However, vector alone- and isoform 3-expressing Tapasin^-/-^ fibroblasts had no detectable TAP protein, presumably because isoform 3 lacks the transmembrane domain necessary for TAP stabilization.

### ERp57 interacts with the Tapasin isoforms

Since all three isoforms of Tapasin retained the N-terminal domain responsible for interactions with ERp57 and other members of the PLC, it was determined whether any physical interaction could be detected between Tapasin and ERp57. Tapasin^-/-^ fibroblasts expressing the Tapasin isoforms or vector alone were lysed, and Western blots or immunoprecipitations were performed to detect ERp57 in association with Tapasin. ERp57 was co-immunoprecipitated and detected by ERp57 antibodies in human Tapasin immunoprecipitated samples (**Figure 3C**, middle panel), indicating the mouse ERp57 interacts with the Tapasin isoforms. The predicted amino acid lengths for Tapasin isoforms are as follows: isoform 1 is 448 amino acids, isoform 2 is 459 amino acids, and isoform 3 is 429 amino acids. These isoforms typically migrate in proportion to their predicted amino acid lengths. However, the similarity in molecular weight between isoform 1 (ER-retained) and isoform 3 (secreted) could stem from post-translational modifications (PTMs) or differences in SDS binding. PTMs like glycosylation or phosphorylation might subtly alter protein mass without significant effects on SDS-PAGE mobility. Additionally, membrane-bound proteins (e.g., isoform 1) may interact differently with SDS, leading to anomalous migration that masks actual differences in weight.

### Rate of ER exit of MHC class I molecules loaded by the Tapasin isoforms

To ascertain any novel functions of isoform 2 compared to isoform 1, a series of assays were employed to assess the quality of the peptides being loaded onto MHC I in Tapasin^-/-^ fibroblasts expressing either isoform. Previous studies have demonstrated that loading of peptides that suboptimally stabilize MHC I can result in different kinetics of ER exit of MHC I molecules, or more rapid turnover of MHC I at the cell surface (25, 31, 33, 36, 37). To address the former point, pulse-chase analysis followed by immunoprecipitation with an antibody (P8) recognizing all forms (both folded and unfolded) of H-2K^b^ was performed. This was followed by Endoglycosidase H (EndoH) digestion to assess the rate of H-2K^b^ exit from the ER of Tapasin^-/-^ fibroblasts expressing each of the Tapasin isoforms or pMX-pie vector alone. H-2K^b^ was immunoprecipitated from cell lysates at various time points up to four hours following a fifteen minute pulse with ^35^S-labeled cysteine and methionine. The rate of acquisition of resistance to Endo H digestion is indicative of passage through the medial Golgi. In Tapasin^-/-^ fibroblasts expressing isoform 3 and vector alone, very little H-2K^b^ acquired EndoH resistance after four hours of chase. At two hours, it appeared that much of the H2K^b^ was being degraded. This indicates that very little H-2K^b^ exited the ER and was likely degraded after a few hours, consistent with flow cytometry experiments which showed almost no surface expression of MHC I in these cells. In contrast, some H-2K^b^ from Tapasin^-/-^ fibroblasts expressing isoform 1 and 2 started acquiring resistance to EndoH digestion after ten minutes of chase. By thirty minutes, approximately half of the labeled H-2K^b^ molecules had acquired EndoH resistance, and by sixty minutes virtually all of the labeled H-2K^b^ had acquired EndoH resistance. The rate of acquisition of EndoH resistance was similar between Tapasin^-/-^ fibroblasts expressing isoform 1 and 2, suggesting that both isoforms mediate similar kinetics of peptide loading and ER exit of this MHC I allele in these cells (**Figure 3E**, left side of graph). Furthermore, these effects were apparent even without the EndoH digestion step. H-2K^b^ is glycosylated and acquires higher carbohydrate structures after leaving the ER, leading to an increase in molecular weight that is evident by decreased mobility in SDS-PAGE gels. This up-shift in molecular weight started to occur by ten minutes of chase in Tapasin^-/-^ fibroblasts expressing isoform 1 and 2, although there was still some lower molecular weight ER-form visible as well, leading to the detection of two bands. The higher molecular weight band is equivalent to the EndoH-resistant band in EndoH-digested samples. This higher molecular weight band constituted the majority of H-2K^b^ in the cells by thirty minutes of chase in cells expressing isoform 1 and 2, and was the only form present thereafter (**Figure 3E**, right side of graph). In contrast, in Tapasin^-/-^ fibroblasts expressing isoform 3 or vector alone, the lower molecular weight form predominated over the four hour chase. A small amount of a higher molecular weight form did appear in both samples after ten minutes, but did not increase in intensity beyond thirty minutes. This is consistent with EndoH-digested samples, in which the majority of H-2K^b^ remains EndoH sensitive in these cells.

### Thermostability of MHC class I molecules loaded by Tapasin isoforms

The decay of H-2K^b^ on the cell surface is another indirect measure of the quality of peptides loaded onto MHC I in the ER. Loading of suboptimal peptides in the ER, which has been observed in Tapasin-deficient cells (31, 33), leads to more rapid turnover and disassembly of the MHC I complex once it reaches the cell surface. In order to assess the stability of surface MHC I, *de novo* MHC I being synthesized and assembled in the ER must be blocked from reaching the cell surface and replenishing the MHC I being turned over. This is achieved by blocking ER to Golgi transport with Brefeldin A (BFA), followed by measurement of surface H-2K^b^ by flow cytometry with a conformation-dependent antibody at various time points after BFA addition at 37°C compared to untreated cells. As shown in **Figure 3D**, Tapasin^-/-^ fibroblasts expressing isoform 1 and 2 had very similar decay curves, suggesting that the peptides loaded by each Tapasin isoform onto H-2K^b^ in the ER of these cells were very similar in their binding affinity and ability to stabilize the H-2K^b^ complex. Surface H-2K^b^ levels on Tapasin^-/-^ fibroblasts expressing isoform 3 and vector alone were too low to assay their stability with this technique.

### Loading of exogenous peptides onto MHC class I by isoform 3

Isoform 3 is unable to mediate MHC I surface expression in Tapasin^-/-^ fibroblasts, likely because of its inability to stabilize the TAP complex due to its lack of the Tapasin transmembrane domain. It was also found that Tapasin^-/-^ fibroblasts expressing this isoform demonstrated a decreased ability to mediate the loading of an exogenously-added H-2K^b^-binding peptide when compared to Tapasin^-/-^ fibroblasts expressing vector alone. Tapasin^-/-^ fibroblasts expressing isoform 1 and 2 did not show any reproducible differences between loading of exogenous peptides onto MHC class I by isoform 3 (data not shown).

**Figure 4:**
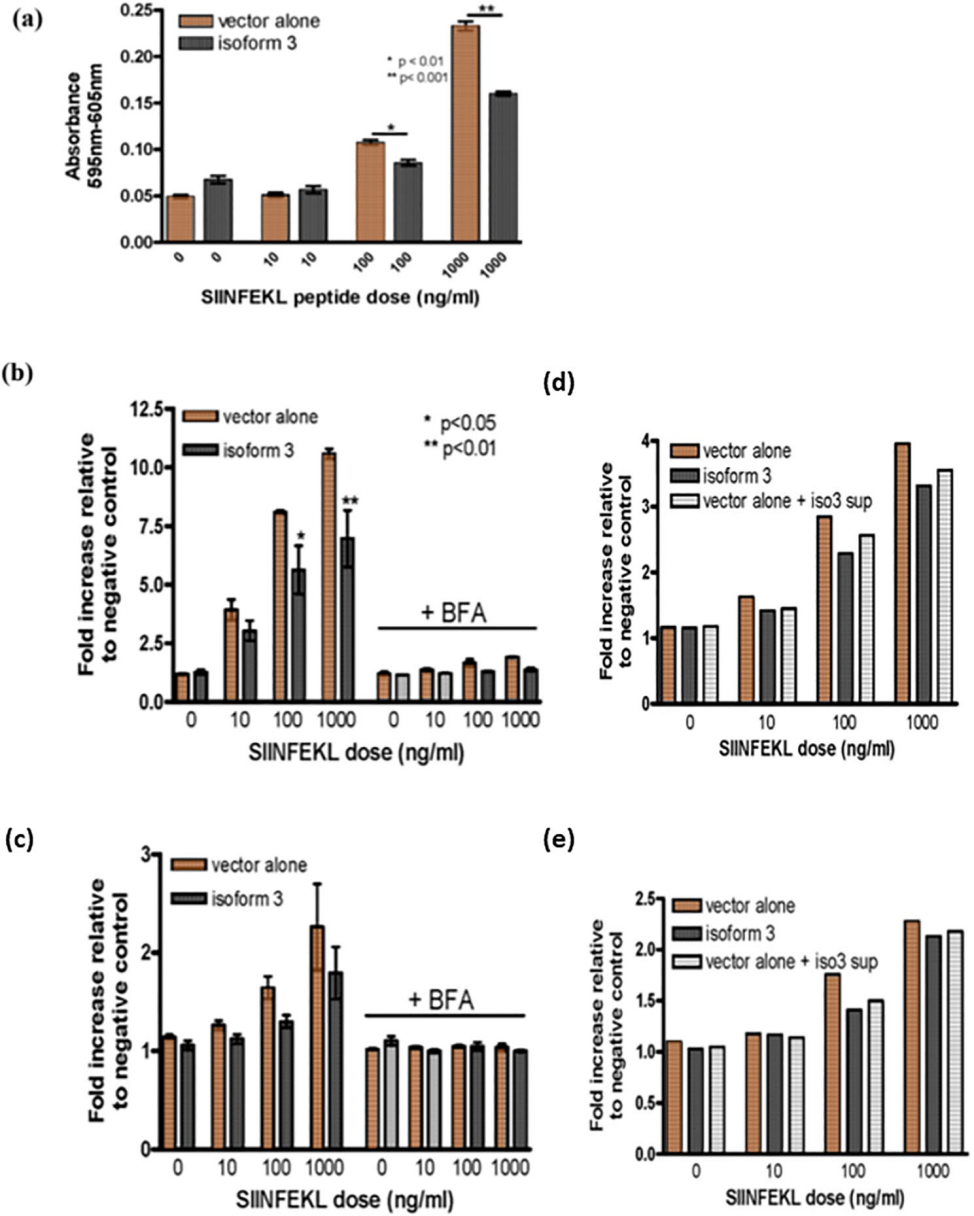
Peptide loading facilitated by different Tapasin isoforms. **(A)** Decreased loading of exogenous peptide loading onto MHC class I and reduced T cell activation in Tapasin-/- fibroblasts expressing Tapasin isoform 3. **(B, C)** Loading of exogenous peptide onto MHC class I and total MHC class I surface levels are decreased in Tapasin^-/-^ fibroblasts expressing isoform 3, and this process is abrogated when the intracellular of MHC class I molecules is inhibited. The trends are apparent at each dose and the asterisks denote the does where this is statically significant. **(D, E)** Soluble isoform 3 decreases the loading of exogenous peptide onto MHC class I in Tapasin^-/-^ fibroblasts. Loading of exogenous SIINFEKL peptide onto H-2K^b^ is decreased in Tapasin^-/-^ fibroblasts expressing isoform 3 compared to vector alone-expressing cells, resulting in decreased activation of B3Z T cells. Tapasin^-/-^ fibroblasts expressing isoform 3 or vector alone were incubated with SIINFEKL at indicated doses for 1 hr at 37°C, washed extensively, fixed and incubated overnight at a 1:1 ratio with B3Z T cells, whose TCR recognizing SIINFEKL in the context of H-2K^b^ leads to the production of β-galactosidase under the control of an NFAT element. Levels of β-galactosidase produced were measured by absorbance at 595 nm with the 605 nm wavelength subtracted with CPRG substrate. **(B, C)** Addition of exogenous SIINFEKL peptide to the media of Tapasin^-/-^ fibroblasts expressing isoform 3 or vector alone results in formation of SIINFEKL/H-2K^b^ peptide complexes **(B)** and total H-2K^b^ levels **(C)** with increasing amounts formed with increasing doses of SIINFEKL. However, Tapasin^-/-^ fibroblasts expressing isoform 3 form fewer SIINFEKL/H-2K^b^ peptide complexes at the cell surface compared to cells expressing vector alone (**A**- left panel) at any given SIINFEKL dose (statistically significant for 100 and 1000 ng/ml doses), as detected by flow cytometry with a SIINFEKL/H-2K^b^-specific antibody 25.D1.16. The formation of these complexes is abrogated by blocking nascent H-2K^b^ trafficking to the cell surface with BFA, that inhibits the trafficking of nascent MHC class I molecules from the endoplasmic reticulum (ER) to the Golgi apparatus, disrupting the formation and surface expression of MHC class I molecules., although isoform 3-expressing cell still have slightly reduced SIINFEKL/H-2K^b^ compared to vector alone under these conditions (**B** - right panel). **(C)** Tapasin^-/-^ fibroblasts expressing isoform 3 also have lower total H2K^b^ surface levels compared to cells expressing vector alone upon addition of exogenous SIINFEKL peptide to the media (c, left panel), but this effect is totally abrogated by addition of BFA and no difference is observed between vector alone and isoform 3 expressing cells (b -right panel). Loading of exogenous SIINFEKL by vector alone-expressing cells pre-incubated overnight with isoform 3 supernatant shows intermediate levels of SIINFEKL/H-2K^b^ complexes **(D)** and total surface K^b^ levels **(E)**, which are in between vector alone and isoform 3-expressing controls, indicating extracellular isoform 3 is likely responsible for the decreased loading of SIINFEKL observed. Data shown represents the results of one flow cytometry experiment, which was repeated three times.

When SIINFEKL, a peptide derived from ovalbumin that binds to H-2K^b^, is added to the media in which the cells are growing, it associates with H-2K^b^ through a peptide exchange mechanism. This association can be detected with the 25.D1.16 antibody, which recognizes H-2K^b^ loaded specifically with the SIINFEKL peptide; the association may also be detected with the B3Z hybridoma T cell line, whose TCR specifically recognizes SIINFEKL peptide-loaded H-2K^b^ and results in the cells producing β-galactosidase under the control of the NF-AT promoter in response to TCR engagement (30). When the amount of H-2K^b^/SIINFEKL staining on Tapasin^-/-^ fibroblasts expressing isoform 3 was compared with Tapasin^-/-^ fibroblasts expressing vector alone by flow cytometry, the isoform 3-expressing cells consistently showed a small but reproducible decrease in fluorescence intensity. This indicates that isoform 3 expressing cells form fewer H-2K^b^/SIINFEKL complexes compared to vector alone expressing cells (**Figure 4B**, left side of graph). This effect was apparent at all three doses of exogenously-added SIINFEKL used in the assay, and is presumably due to an inhibitory function of isoform 3 on SIINFEKL loading/exchange onto H-2K^b^ that is absent in vector alone-expressing cells. This phenomenon can be explained by differences in how isoform 3 influences H-2K^b^ peptide loading and surface trafficking. Isoform 3 may reduce peptide loading efficiency in the ER, leading to fewer SIINFEKL/H-2K^b^ complexes at the cell surface compared to vector-only cells. The lower total H-2K^b^ surface levels in isoform 3-expressing cells upon SIINFEKL addition could reflect inefficient recycling or stabilization of H-2K^b^. The equalized levels upon BFA treatment suggest isoform 3 specifically affects nascent H-2K^b^ trafficking, rather than overall surface retention of pre-existing H-2K^b^ molecules.

In addition, the number of total H-2K^b^ complexes on the cell surface of SIINFEKL-incubated cells was detected with a conformation-dependent antibody recognizing H-2K^b^ regardless of the specific peptide loaded. Total surface H-2K^b^ levels were virtually undetectable without the addition of peptide, as seen in **Figure 2**. However, addition of exogenous SIINFEKL peptide at higher doses did lead to an increase in total H-2K^b^ levels. This effect is presumably due to exchange of low affinity self-peptides for the high affinity SIINFEKL peptide, allowing cell-surface expression of H-2K^b^ through stabilization of the trimeric heavy chain/β2-microglobulin/peptide complex. When a low affinity peptide is loaded onto MHC-I in the ER, the complex may reach the cell surface but is unstable and rapidly dissociates; however, a high affinity peptide prolongs the half-life of the complex and allows detection by flow cytometry with the conformation-specific antibody. Isoform 3-expressing cells also had lower levels of total H-2K^b^ on the cell surface compared with vector-alone (VA) expressing cells following incubation with any given dose of exogenous SIINFEKL (**Figure 4C**, left side of graph). This also supports the hypothesis that isoform 3 has an inhibitory effect on peptide exchange.

**Figure 5:**
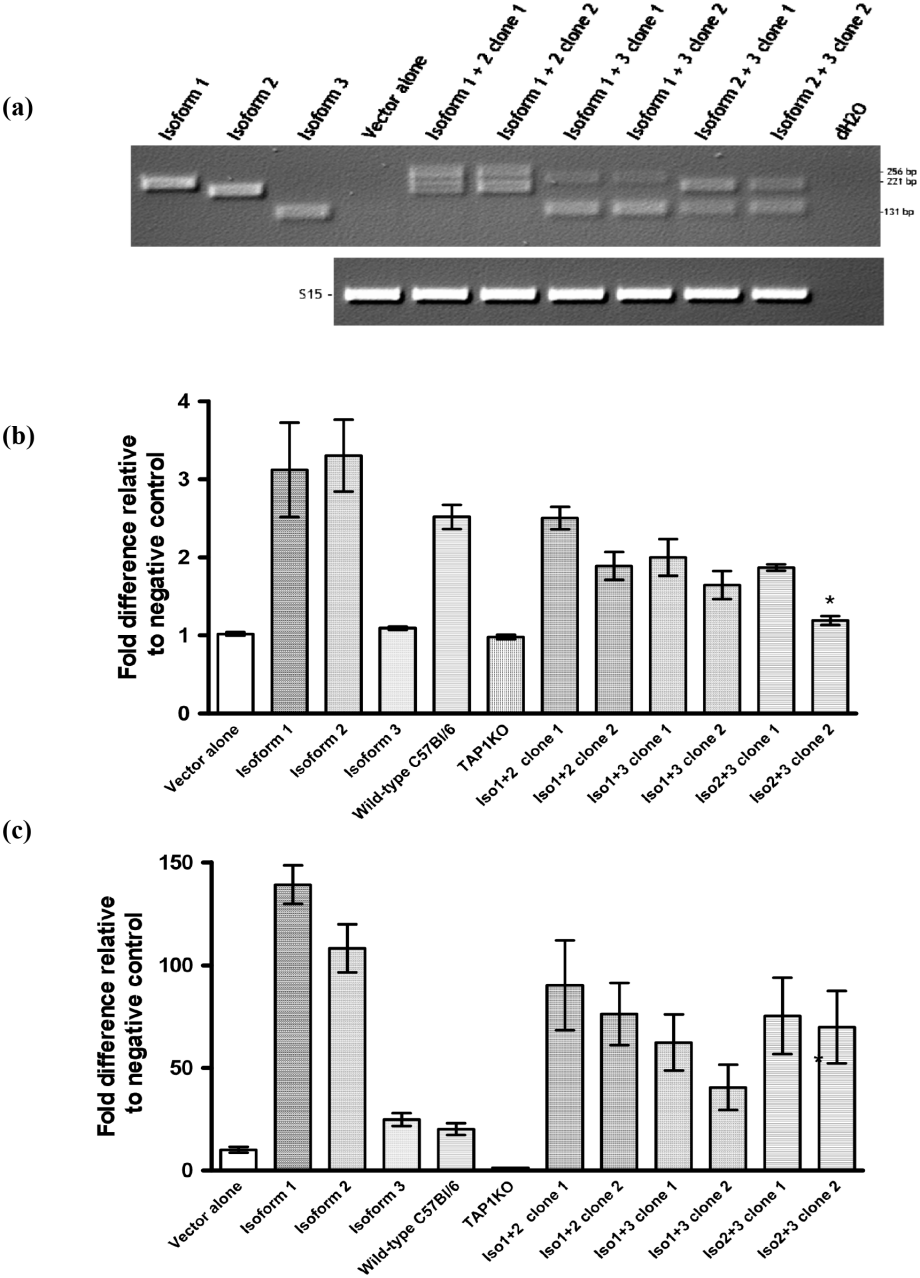
Co-expression of Tapasin isoforms. **(A)** Generation and confirmation of Tapasin isoform-expressing Tapasin-/- fibroblasts by recombinant retrovirus co-infection and PCR screening. **(B, C)** Co-expression of multiple Tapasin isoforms reduces surface MHC class I expression compared to single isoform expression. Tapasin^-/-^ fibroblast clones expressing different combinations of Tapasin isoforms were generated by co-infection with mixtures of recombinant retroviruses. Expression of Tapasin isoforms in the transfected clones was confirmed by PCR, with amplification of specific isoform sequences calibrated to a control PCR using the S15 housekeeping gene as a loading control. This screening method allowed for the selection of clones with stable isoform expression for subsequent functional analyses. Data shown represent the results of at least two independent experiments. Surface MHC class I expression levels on Tapasin-transfected fibroblasts expressing combinations of isoforms was determined. Surface MHC class I expression levels for both H-2K^b^ **(B)** and H-2D^b^ **(C)** alleles were evaluated on Tapasin-/- fibroblasts expressing combinations of Tapasin isoforms using flow cytometry. Results indicate that expression of multiple Tapasin isoforms led to decreased surface MHC class I expression compared to cells expressing a single isoform. Notably, only clone 2 of the isoform 2 + 3 combination exhibited statistically lower H-2K^b^ surface levels compared to cells expressing isoform 1 or isoform 2 alone (**A**, *p<0.01). These data suggest that specific isoform combinations may influence the surface expression of MHC class I molecules, with certain combinations resulting in reduced expression levels. The data were compiled from at least three independent experiments, ensuring reproducibility of the findings.

Furthermore, the H-2K^b^/SIINFEKL complexes generated by the addition of exogenous peptide were able to stimulate B3Z T cells. Consistent with the flow cytometry experiments, isoform 3-expressing cells demonstrated a reduced ability to activate B3Z T cells at a given dose of exogenous SIINFEKL compared to vector alone expressing cells (**Figure 5**). These experiments also demonstrate that the H2K^b^/SIINFEKL complexes generated by addition of exogenous SIINFEKL peptide to the media are functional in their ability to stimulate NF-AT activation in T cells.

It was further investigated whether the observed loading of exogenous SIINFEKL was mainly due to peptide exchange at the cell surface, or required trafficking of nascent H-2K^b^ molecules from the ER. ER to Golgi transport was blocked by pre-treating Tapasin^-/-^ fibroblasts expressing isoform 3 or vector alone with Brefeldin A (BFA) prior to adding exogenous SIINFEKL. Only very small amounts of H-2K^b^/SIINFEKL complexes formed on the cell surface of both isoform 3 and vector alone-expressing BFA-treated fibroblasts, though the amount formed was again slightly higher in vector alone expressing cells compared to isoform 3-expressing cells (**Figure 4B**, right side of graph). BFA treatment also completely abrogated the increase in total H-2K^b^ observed previously in both isoform 3 and vector alone-expressing cells to an equal degree (**Figure 4C**, right side of graph). Increasing doses of SIINFEKL resulted in only very small increases of H-2K^b^/SIINFEKL complexes on BFA-treated cells and did not appreciably affect total H2K^b^ levels. This suggests that a small number of H-2K^b^ already present at the cell surface of these cells may have exchanged intracellularly-loaded, low affinity peptides for SIINFEKL at the cell surface. However, due to the inability for further H-2K^b^ molecules to reach the cell surface from the ER, the number of H-2K^b^/SIINFEKL complexes remained static and were close to saturation with SIINFEKL even at the lower doses of SIINFEKL added. Therefore, it appears that ER to Golgi transport of nascent MHC I molecules is important for the larger amounts of exogenous SIINFEKL loading observed in the absence of BFA.

To assess whether intracellular or extracellular isoform 3 is responsible for reducing the formation of SIINFEKL/H-2K^b^ complexes, supernatant from isoform 3-expressing Tapasin^-/-^ fibroblasts was transferred to vector alone-expressing fibroblasts and incubated overnight prior to addition of increasing doses of SIINFEKL. Both SIINFEKL/H-2K^b^ complex formation (**Figure 4D**) and total H-2K^b^ surface expression (**Figure 4E**) appeared to be intermediate between isoform 3 and vector alone-expressing controls. This suggests that part of the inhibitory effect of isoform 3 on SIINFEKL loading may be from the extracellular form but that full effects require either intracellular expression of isoform 3 or more consistent synthesis from within the cell, as the half-life of isoform 3 in the media is unknown.

Together these results suggest that isoform 3 may actively suppress the exchange and loading of exogenously-added peptides onto the H-2K^b^ MHC I allele. This could form part of a mechanism that evolved to avoid potential killing of bystander cells located close to pathogen-infected cells that might bind pathogenic peptides released from infected cells and thereby be targeted for CTL-mediated killing.

### Expression of combinations of isoforms in Tapasin^-/-^ fibroblasts

Since all cells, cell lines and tissues examined naturally express isoform 1, isoform 2 and in some cases isoform 3, the effect of expressing the different isoforms in combinations was evaluated in Tapasin^-/-^ fibroblasts to observe any synergistic effects on MHC I expression, including potential dominant negative effects (particularly for isoform 3). Tapasin^-/-^ fibroblasts were infected with a 1:1 mixture of recombinant retroviruses encoding isoform 1 + isoform 2, isoform 1 + isoform 3, isoform 2 + isoform 3, or all three isoforms, which were then selected in puromycin, and individual clones were sorted by FACS based on GFP expression and expanded in culture. Clones were then screened for expression of more than one isoform. The majority of cells had only been infected with one of the retroviruses and expressed only one isoform. No clones were identified that expressed all three isoforms. However, two clones each were identified that expressed each combination of two isoforms (**Figure 5A**).

### Individual clones for expression of both Tapasin isoform mRNAs by RTPCR

The H-2K^b^ and H-2D^b^ surface expression of the clones expressing combinations of isoforms were evaluated by flow cytometry (**Figures 5B, C**). Although expression levels varied between each pair of clones, none were statistically different from one another. Overall, it appeared that in general, expression of more than one isoform resulted in slightly lower MHC I surface expression compared to cells expressing either isoform 1 or 2 alone. Only clone 2 of the isoform 2 + 3 combination had statistically lower H-2K^b^ surface expression compared to cells expressing either isoform 1 or isoform 2 alone, or wild-type C57BL/6 fibroblasts. H-2D^b^ expression on isoform 1 + 3 clone 2 was statistically lower than cells expressing isoform 1 alone, but was not statistically different than cells expressing isoform 2 alone. However, since both these differences were seen in only one clone from each pair, and only in one of the two MHC I alleles, it was hypothesized that the differences might be due to positional effects in the site of retroviral gene insertion rather than an effect from the genes themselves. A true gene-specific effect would have been mirrored by both clones of each group. Therefore, combinations of isoforms did not appear to exhibit any dominant negative or other synergistic effects in this cell line.

### Isoform expression in the DC2.4 cell line

**Figure 6:**
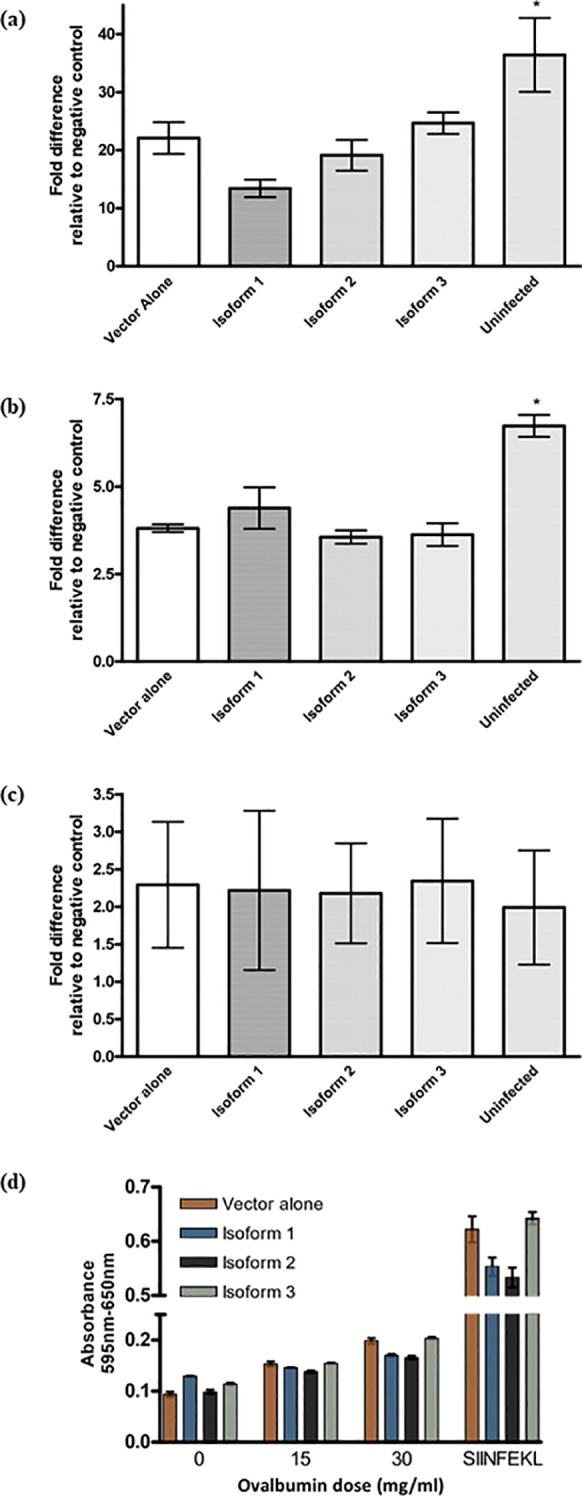
The effect of Tapasin isoforms on MHC class I surface level. **(A–C)** Expression of Tapasin isoforms alters MHC class I surface levels in a wild-type dendritic cell line but does not affect MHC class II expression. **(D)** Cross-presentation and cross-priming of soluble ovalbumin by a wild-type dendritic cell line is not affected by any of the three Tapasin isoforms. Recombinant retroviruses were used to express each isoform or vector alone in the DC2.4 cell line. Transduced cells were selected in puromycin and sorted to ensure equal GFP expression across populations. MHC class I surface expression was assessed by flow cytometry, evaluating H-2K^b^ (**Figure 4**.12a) and H-2D^b^ alleles. Data reflect bulk-sorted populations. Flow cytometry analysis of DC2.4 mouse dendritic cell lines stably infected with recombinant retroviruses expressing Tapasin isoforms, vector alone, or uninfected controls showed that expression of Tapasin isoforms did not significantly affect MHC class II **(B)** or H-2D^b^ **(C)** surface expression. However, H-2K^b^ levels **(A)** were slightly reduced in cells expressing isoforms 1 and 2 compared to the vector-alone and isoform 3-expressing cell lines, though these differences were not statistically significant. Additionally, surface MHC I expression was significantly lower in all transduced cell lines compared to uninfected cells, suggesting that the retroviral transduction itself may reduce surface MHC class I levels. Data were compiled from at least three independent experiments. **(D)** DC2.4 dendritic cells expressing Tapasin isoforms or vector alone were incubated overnight with increasing doses of soluble ovalbumin, washed to remove excess peptide, and then co-cultured with B3Z T cells at a 1:1 ratio to assess the cross-presentation of SIINFEKL peptides. Cross-presentation efficiency and T cell activation were similar across all conditions, showing no statistically significant differences between the expression of Tapasin isoforms and vector alone. Cross-presentation and cross-priming of soluble ovalbumin by DC2.4 expressing the Tapasin isoforms or vector alone increases with increasing doses of ovalbumin but is unaffected by isoform expression. These results suggest that expression of any of the three Tapasin isoforms does not affect the ability of DC2.4 cells to cross-present soluble ovalbumin. Data were compiled from three independent experiments.

Another way to address potential synergistic effects between isoforms was to express the isoforms in cells already expressing mouse Tapasin, which is very similar to human isoform 1 in structure and function. The transformed dendritic cell line DC2.4 was selected because it also allowed for the evaluation of the isoforms’ effect on MHC II surface expression and on cross-presentation of exogenous antigens on MHC I, as these are thought to occur only in professional antigen presenting cells such as DCs and not in fibroblasts (38). Recombinant retroviruses were used to express each isoform or vector alone in the DC2.4 cell line, followed by selection in puromycin and sorting for cells expressing equal levels of GFP using flow cytometry. The MHC class I surface expression of the transduced DC2.4 cells were evaluated by FACS for both H-2K^b^ (**Figure 6A**) and H-2D^b^ alleles (**Figure 6B**). Interestingly, the effect of Tapasin isoform expression on H-2K^b^ in DC2.4 cells seemed to be the opposite of the effect of the isoforms in Tapasin^-/-^ fibroblasts: DC2.4 expressing isoform 1 and 2 appeared to have lower surface H-2K^b^ compared to cells expressing isoform 3 and vector alone; however, these differences were not statistically significant (**Figure 6A**). H-2D^b^ levels were unaffected by any expression of the isoforms compared to vector alone (**Figure 6B**). Interestingly, all transduced cells had statistically significantly lower expression of H-2K^b^ and H-2D^b^ compared to uninfected DC2.4, suggesting the retroviral transduction may have caused an overall decrease in MHC I surface expression. This effect did not appear to extend to MHC II expression, however. MHC II expression varied considerably between assays, but statistically there was no significant effect from any isoform or vector alone expression on overall levels of MHC class II surface expression (**Figure 6C**). Flow cytometry shows MHC class II and H-2D^b^ levels were unaffected by expression of the Tapasin isoforms versus vector alone (**Figures 6B, C**). H-2K^b^ levels were slightly reduced in DC2.4 expressing isoform 1 and 2 compared to vector alone- and isoform 3-expressing cell lines, but these differences were not statistically significant (**Figure 6A**). Surface expression of both MHC class I alleles was statistically higher in uninfected cells compared to cells infected with any of the recombinant retroviruses (**Figures 6A, B**), suggesting that transduction itself reduced surface MHC class I levels.

### Cross-presentation of soluble ovalbumin by DC2.4 expressing Tapasin isoforms

To evaluate the potential contribution of the Tapasin isoforms to exogenous antigen cross-presentation, soluble ovalbumin at various concentrations was added overnight to DC2.4 expressing the Tapasin isoforms. The following day, excess ovalbumin not internalized by the cells was washed off, cells were gently fixed to halt MHC class I turnover and nascent MHC I loading, and the cells were mixed with B3Z to assess the amount of H-2K^b^/SIINFEKL generated. All cells presented increased amounts of H-2K^b^/SIINFEKL with increasing amounts of ovalbumin added, as expected; however, expression of the isoforms did not have an appreciable effect on the ability of DC2.4 to cross-present exogenous ovalbumin-derived peptides on H-2K^b^ (**Figure 6D**). At each given dose of ovalbumin, there was no statistically significant difference between the cells expressing the different isoforms though the increases observed with the highest dose of ovalbumin were statistically significant. At higher ovalbumin doses, it appeared that isoform 1 and 2-expressing cells expressed fewer H2K^b^-SIINFEKL complexes compared to isoform 3 and pMX-pie vector-alone expressing cells; however, this is also consistent with the slightly lower overall H-2K^b^ surface expression levels on these cells seen earlier in flow cytometry experiments (**Figure 6A**) and is further supported by the fact that pulsing cells with exogenous SIINFEKL (as a positive control) resulted in lower B3Z activation by isoform 1 and 2-expressing cells compared to pMX-pie vector alone and isoform 3.

## Discussion

Studies reveal that Tapasin functions as a critical chaperone for MHC class I molecules by altering their structure to favor a peptide-receptive state. Through widening the peptide-binding groove, Tapasin enhances the rate of peptide exchange, optimizing the presentation of stable, high-affinity antigenic peptides on MHC class I molecules. This peptide editing process depends on the disruption of conserved hydrogen bonds at the groove’s C-terminal and specific interactions throughout the binding groove, which work together to selectively exclude peptides unable to fully disengage Tapasin (39). This selective mechanism not only sharpens the antigenic peptide repertoire but also involves Tapasin’s association with ERp57, a thiol oxidoreductase that stabilizes MHC I molecules, ensuring they are primed for high-quality peptide binding. Beyond intracellular antigen presentation, alternative protein isoforms of Tapasin may support cross-presentation pathways in specialized immune cells like dendritic cells, allowing exogenous antigens to be presented on MHC I molecules, which broadens immune surveillance potential. Through these multifaceted roles, Tapasin refines immune surveillance and enhances immune tolerance, making it a potential therapeutic target in autoimmune diseases and immunotherapies (40).

In our studies, the ability of Tapasin isoforms 1 and 2 to mediate classical endogenous MHC class I surface expression and presentation of viral peptides to CTLs in Tapasin^-/-^ fibroblasts appears to be directly linked to their ability to stabilize TAP1 and TAP2 at the protein level (**Figures 2**–**4**). The regions of TAP that interact with Tapasin are the N-terminal extensions of the core multiple membrane spanning domains common to all ABC transporters (41–43). The regions of Tapasin responsible for mediating interactions with TAP have also been well characterized and consist mainly of residues within the Tapasin transmembrane domain (34, 35). These detailed studies have contributed a great deal to our understanding of how Tapasin stabilizes the TAP heterodimer, and since both isoform 1 and 2 encode the transmembrane domain responsible for this function, it is expected that these isoforms stabilize TAP and isoform 3, which does not contain any of these critical TAP-stabilizing regions, does not. However, this does make it very difficult to ascertain and/or separate additional novel functions of the Tapasin isoforms for isoform 3, since the function of Tapasin is inextricably linked to the presence of TAP, and any potential subtle differences might be undetectable in its absence. Thus, we must rely more heavily on tissue distribution and expression data of the Tapasin isoforms to understand their function.

The finding that ERp57 co-immunoprecipitates with all three Tapasin isoforms (**Figure 5**) suggests that the Tapasin isoforms may mediate assembly of a complete and functional peptide-loading complex, including other members such as calreticulin and MHC I. Indeed, Tapasin has been proposed to require covalent linkage to ERp57 for proper function (44). This could indicate that PLC assembly occurs in the non-ER compartments where Tapasin isoforms have been found to localize in small amounts, such as TGN and lysosomes. While isoform 3 is not likely to associate with TAP, its association with ERp57 could mean that it too acts as a scaffold for PLC assembly, though it remains unknown whether the secreted isoform also interacts with ERp57 and other PLC components. It is also unknown if a soluble PLC complex in the absence of TAP association would function in a different manner than TAP-associated PLCs.

It was important to ascertain whether isoform 1 and 2 mediated different effects on MHC I antigen presentation beyond their common ability to stabilize TAP. Tapasin is known to influence the repertoire of peptides presented by MHC I molecules, but eluting and sequencing peptides bound by surface MHC I molecules loaded by the different isoforms remains uninvestigated. Instead, we began by employing more indirect assays to assess the quality of peptides being loaded by the different isoforms onto H-2K^b^. It was hypothesized that the nature of the peptides loaded might differ if the isoforms altered the peptide-binding preferences of the MHC I molecules in distinct ways. Other studies have shown that suboptimally-loaded MHC I molecules, such as those from cells of transgenic animals deficient in various APM components like ERp57 (45) and Tapasin itself (25, 31), exit the ER at faster rates and have decreased cell surface thermostability at 37°C. Suboptimal peptide loading leading to thermally unstable surface MHC I can have serious consequences in terms of generating CD8 T cell-mediated immune responses, as the MHC I molecules might disassemble and turn over prior to interacting with CD8 T cells for periods long enough to generate and/or sustain an effective immune response. However, both ER exit rates and cell surface thermostability of H-2K^b^ molecules appeared very similar in Tapasin^-/-^ fibroblasts expressing isoform 1 and isoform 2 (**Figures 3D, E**), suggesting that the loaded peptides possessed similar abilities to stabilize this MHC I allele. Nevertheless, it is known that different MHC I alleles, particularly in humans, possess greatly varying dependencies on Tapasin for optimal peptide loading, with some alleles such as HLA-B8 and A1 being highly dependent on Tapasin, and others such as HLA-B7, A2 and A3 being virtually unaffected by the presence or absence of Tapasin (46–48). Since the assay used here examines human Tapasin isoforms in conjunction with a single mouse MHC I allele, H-2K^b^, it is possible that this allele may be among those that are not very dependent on Tapasin for optimal peptide loading. The dependencies of mouse MHC I alleles on Tapasin have been less well studied than human MHC I alleles, and the degree of influence Tapasin exerts on H-2K^b^ specifically has not been studied extensively. However, the low surface expression of H-2K^b^ in Tapasin^-/-^ mice on a C57BL/6 background does suggest it has a Tapasin-dependent phenotype. Nevertheless, it remains possible that differences might be observed between isoform 1 and 2 if loading of a different MHC I allele were tested.

It was initially somewhat surprising that no effect on MHC I from soluble Tapasin isoform 3 was observed in the Tapasin^-/-^ fibroblasts, since another group reported a very similar recombinant soluble form of Tapasin that did enhance MHC I loading in cells lacking Tapasin (37). Lehner et al. demonstrated that a recombinant soluble form of human Tapasin created by deleting the transmembrane and cytoplasmic regions could still reconstitute MHC I surface expression in 721.220 cells stably expressing various HLA molecules, even though the soluble Tapasin no longer associated with TAP (37). The obvious difference between the Lehner et al. study and this study it that we are studying a naturally occurring soluble form of Tapasin while the previous study studied a recombinant form of Tapasin that does not occur naturally. A study by Gao et al. (36) identified a novel isoform of human Tapasin that retains introns 5, 6 and 7, resulting in the expression of a truncated Tapasin protein due to the introduction of a new stop codon that terminates translation immediately prior to the transmembrane domain. This truncated Tapasin protein was predicted to be soluble, as it retained the ER signal peptide and Tapasin N-terminal domain but contained 8 novel amino acids at its C terminus and lacked an ER retention motif. When this isoform was expressed in 721.220 cells stably expressing HLA-B8, it was also capable of restoring MHC I antigen presentation despite a lack of binding to TAP. However, the peptides loaded were found to have suboptimal affinity for MHC I compared to those loaded by wild-type Tapasin (36). Both these studies were undertaken in the human Tapasin-deficient 721.220 cell line transfected with HLAB8 (or other HLA alleles) rather than the mouse Tapasin^-/-^ fibroblast line used in this study. Unlike the murine Tapasin^-/-^ cells, 721.220 cells express large amounts of TAP since they have been transformed by EBV; TAP levels in EBV-expressing cells are known to be much higher compared to EBV-negative cells, in which TAP is more limiting (37, 49). It is likely true that Tapasin expression in EBV-transformed cells is higher than in uninfected cells as well. Furthermore, the 721.220 cells do still produce a N-terminally truncated form of Tapasin that localizes in small amounts to the ER and contains the TAP-stabilizing transmembrane domain, which is likely sufficient to stabilize TAP protein expression (50). Therefore, it is difficult to compare our studies to the studies done in 721.220, as clearly these cells are not completely Tapasin (and TAP) deficient. It would be very interesting to express isoform 3 in these cells; however, the 721.220 cells stably transfected with the otherwise-deleted HLA genes were not available for this study, and many cloned HLA molecules (which could have been used to generate our own stable transfectants) appear to be proprietary and were similarly inaccessible.

The existence of isoform 3 suggests that it must have a function distinct from stabilizing TAP, and certainly there are many other different functions ascribed to Tapasin (33, 51–54). Two approaches were tried to ascertain the function of isoform 3: 1) addition of a known H-2K^b^-binding peptide, SIINFEKL, to the extracellular media, thereby bypassing the need for peptide import into the ER by TAP, and 2) expression of isoform 3 in combination with the other two TAP-stabilizing Tapasin isoforms to see if there was any synergistic or dominant–negative function for isoform 3 that might be revealed upon restoration of TAP protein expression by the other isoforms. For the latter experiments the Tapasin isoforms were expressed in combinations (isoform 1 + 2, isoform 2 + 3 and isoform 1 + 3) in Tapasin^-/-^ fibroblasts, and also each isoform was expressed individually in DC2.4 cells that have endogenous mouse Tapasin capable of stabilizing TAP, to look for any combinatorial effects (**Figures 5B, C**, **6**). In both cell lines, no clear dominant negative effects of isoform 3 were observed on the surface levels of either H-2K^b^ or H-2D^b^. In fact, it appeared that the expression of multiple Tapasin isoforms led to a decrease in overall surface MHC I expression compared to cells transduced with only one isoform, regardless of the specific isoform combinations expressed. This may indicate that elevated Tapasin expression results in more stringent editing functions in the ER, leading to an overall decrease in surface expression as more MHC I molecules remain in the ER. One clone (isoform 2 + 3, clone 2) was statistically different from cells expressing either isoform 1 or 2 alone. However, a second clone (clone 1) expressing the same two isoforms did not have any significant decrease in surface MHC I. Therefore, the decreased surface MHC I observed could be due to positional effects from the site of integration of the retroviral constructs rather than an effect from the genes themselves, which should have been apparent in all clones examined. It would be useful to study larger numbers of clones to draw further conclusions as to any combinatorial effects. However, having two different retroviruses infect the same cell is a rare occurrence, and would require the screening of many more clones. No effects on MHC II surface levels were observed in DC2.4 transductants (**Figures 6A-C**), suggesting the novel isoforms do not influence basal MHC II presentation in these cells. It has been proposed that the MHC II chaperone invariant chain can bind and direct MHC I molecules (5, 55, 56) but from the data presented here it appears that the reverse, Tapasin chaperoning MHC II, does not occur.

This study identifies a novel function of Tapasin isoform 3, distinct from classical Tapasin, in selectively regulating MHC I-peptide loading and presentation, particularly for exogenous peptides. In Tapasin-deficient fibroblasts, cells expressing isoform 3 demonstrated significantly reduced formation of H-2K^b^/SIINFEKL complexes at the cell surface compared to vector-only controls (**Figures 4A-C**). This observation was confirmed through both antibody detection and T cell activation assays, indicating that isoform 3 affects the stability or efficiency of peptide loading onto MHC I molecules, likely through a unique chaperone-like action that diverges from the canonical ER-resident Tapasin pathway. Notably, although overall levels of surface H-2K^b^ were similarly low prior to SIINFEKL peptide addition (**Figure 4A**) isoform 3-expressing cells displayed even lower H-2K^b^ levels after peptide incubation relative to vector-alone controls, as detected by antibodies specific to properly folded MHC I (57). This finding suggests that isoform 3 may hinder stable loading of SIINFEKL onto MHC I in association with endogenous β2-microglobulin, rather than through reassembly with bovine β2-microglobulin present in the media (58). Moreover, nearly all SIINFEKL loading was blocked by Brefeldin A (BFA) (**Figures 4B, C**), supporting the conclusion that exogenous peptide loading requires newly synthesized MHC I molecules trafficking from the ER.

Furthermore, studies transferring supernatants from isoform 3-expressing cells to vector-only cells showed partial inhibition of SIINFEKL loading onto H-2K^b^, though this effect was weaker than in cells actively synthesizing isoform 3 (**Figures 4D, E**). These results underscore that isoform 3 may influence MHC I peptide loading through secreted factors or by affecting other compartments, such as the Golgi or lysosomes, if components necessary for MHC I assembly and peptide loading co-localize in these compartments. Thus, isoform 3 appears to play a selective role in refining the antigenic peptide repertoire presented by MHC I, with potential implications for immune surveillance and the prevention of autoimmunity.

Tapasin isoform 3 may play a regulatory role in selectively blocking some exogenous peptides from binding to MHC I molecules, akin to how the invariant chain (CD74) prevents MHC II from premature peptide binding before reaching lysosomal compartments. This selective blocking could help protect uninfected bystander cells from being mistakenly targeted by CTL during viral infections, preventing viral peptides from neighboring infected cells from binding to MHC I on these uninfected cells. By ensuring that MHC I molecules bind peptides only within specific cross-presenting compartments, isoform 3 may aid in precise immune surveillance, reducing the risk of autoreactivity, especially against self-peptides on dendritic cells (DCs) in lymph nodes. This provides further evidence for a timing and compartment-specific function akin to CD74 in the MHC II pathway. Together, these findings suggest that isoform 3 could play a key role in the spatial and temporal regulation of MHC I antigen presentation, fine-tuning immune responses while minimizing unnecessary or harmful CTL activation.

In this study, we found that the isoforms did not significantly influence the cross-presentation of the ovalbumin-derived peptide SIINFEKL onto H-2K^b^ molecules in the DC2.4 cell line (**Figure 6D**). Several explanations may account for this observation. First, despite their high expression levels in mature dendritic cells (DCs) and localization to endo/lysosomal compartments, the isoforms may not participate in antigen cross-presentation and could serve different functions. The complexity of the cross-presentation process, with numerous proposed models and ongoing debates regarding the molecular mechanisms, leaves open the possibility that the isoforms could affect cross-presentation when tested with different antigens or in other DC subsets. For instance, soluble ovalbumin is known to be cross-presented via the mannose receptor (59), while different uptake methods, such as phagocytosis of particulate antigens or endocytosis of immune complexes, may activate distinct mechanisms (60). Previous research has shown that the same antigen can be processed through different pathways based on its form, whether soluble or particulate (61). However, few specific reagents exist for monitoring the cross-presentation of other antigens, complicating these analyses.

Another consideration is that the isoforms’ effects on cross-presentation may be restricted to specific DC subsets not represented by the DC2.4 cell line. Various DC subsets have unique characteristics, locations, and functions (62, 63), and while all can capture and present antigens to naïve T cells, their efficiencies vary. For example, conventional CD8⁺ myeloid DCs (cDC1s) remain the most proficient at cross-presentation (64). Although skin Langerhans cells (65, 66) and plasmacytoid DCs (67–70) have been implicated in cross-priming CD8+ T cells, more recent studies suggest that their contribution is limited compared to cDC1s. Additionally, inflammatory monocyte-derived DCs and certain cDC2 subsets can cross-present under specific conditions. While many DC subsets remain challenging to culture and manipulate *in vitro*, advances in *ex vivo* differentiation techniques and gene-editing approaches have improved our ability to study their functions.

The influence of the isoforms could also be obscured by the presence of endogenous mouse Tapasin in the DC2.4 cell line. For instance, the impact of isoform 3 on reducing the loading of exogenous SIINFEKL peptides in Tapasin-deficient fibroblasts was not easily observable in DC2.4 cells, likely due to the masking effect of endogenous Tapasin. Efforts to express the isoforms in DCs derived from Tapasin-deficient mice encountered technical challenges that hindered our ability to assess their cross-presentation functions. Furthermore, the lack of TAP protein expression in these cells, due to the absence of Tapasin’s transmembrane domain, further complicated our analysis. TAP is not strictly required for loading exogenously-derived peptides in lysosomal cross-presentation models, but it is likely essential for transporting MHC I molecules, complexed with high affinity peptides to the cell surface for subsequent internalization and interaction with exogenous antigens in lysosomal compartments. In the absence of TAP in the endoplasmic reticulum (ER), peptide-deficient MHC I molecules are likely retained, which can significantly impair the efficiency of cross-presentation. The inability to properly load MHC I with peptides hinders their transport to the cell surface, reducing the capacity of antigen-presenting cells to initiate effective T cell responses. Studies have shown that TAP is essential for the optimal loading of peptides onto MHC I molecules, and its absence can lead to decreased surface expression of stable peptide-MHC complexes (71–74). This retention mechanism ultimately affects the cross-presentation pathway, reducing the immune system’s ability to respond to extracellular antigens effectively (5, 8, 9).

In interpreting our findings regarding isoform 3, it is essential to consider additional mechanisms that may elucidate the observed data, particularly focusing on extracellular vesicles (EVs), trogocytosis, and surrogate antigen processing. EVs also known as secreted vesicles, are small membrane-bound particles released by cells that play crucial roles in intercellular communication. They carry various biomolecules, including proteins, lipids, and RNA, which can influence the behavior of recipient cells (75). Given their role in modulating immune responses, EVs may represent a mechanism by which isoform 3 affects antigen presentation, potentially facilitating the transfer of antigens and immune-modulating factors that enhance cross-presentation and T cell activation (75).

Trogocytosis is a process whereby dendritic cells (DCs) and other immune cells extract membrane fragments, including MHC class I/peptide complexes, from neighboring cells. This mechanism enhances antigen acquisition and facilitates the formation of immune synapses, ultimately improving the efficiency of cross-presentation. Research indicates that the trogocytosis of MHC class I complexes from tumors or infected cells significantly enhances DC cross-presentation, leading to improved activation of CD8+ T cells and promoting adaptive T cell responses (9). This phenomenon exemplifies antigenic spread and “antigen cross-dressing”, where immune cells acquire antigens from multiple sources, underscoring the importance of direct cell-to-cell interactions in immune surveillance. Furthermore, Tapasin’s influence on MHC I loading may further modulate the efficiency of antigen presentation, enhancing immune function.

In this regard, the concept of surrogate antigen processing is particularly relevant to our study. This mechanism indicates that antigenic peptides can be secreted in a TAP-dependent manner and subsequently reintroduced onto cells for presentation on MHC class I molecules (12). This process highlights the potential for extracellular peptides, including those contained in EVs, to be recaptured and presented by antigen-presenting cells (APCs), thereby enhancing the diversity of antigens available for recognition by CTL.

In addition, the bystander CTL killing effect refers to a phenomenon in which immune responses are activated against specific antigens, leading to the recognition and destruction of adjacent, uninfected cells that present similar antigens (76). This can occur during infections or vaccinations, where the activation of cytolytic T cells against one antigen inadvertently enhances the immune response against other antigens present in the vicinity, potentially leading to tissue damage. Tapasin Isoform 3 could reduce or modify this effect.

In a model to explain the role of isoform 3 in inhibiting the loading of exogenous peptides onto MHC class I molecules at the cell surface it follows that, first, during the normal antigen presentation pathway, MHC class I molecules are synthesized and loaded with peptides in the endoplasmic reticulum (ER), facilitated by the peptide-loading complex, which ensures high-affinity peptide binding. However, some MHC class I molecules may escape the ER without a peptide or with a low-affinity peptide, leading to unstable complexes that are prone to acquiring exogenous peptides at the cell surface. At the cell surface, isoform 3 could bind to MHC class I molecules that have incomplete or suboptimal peptide cargo, stabilizing them in a conformation that resists further peptide exchange. This ensures that exogenous peptides, which may not represent intracellular processes, are not presented to T cells. The model also raises the possibility that isoform 3 could act as a competitive inhibitor by modifying the peptide-binding site itself. Functionally, this mechanism maintains the fidelity of antigen presentation, avoiding immune evasion by pathogens that might exploit external peptide sources and reducing the risk of autoimmunity by preventing the presentation of self-derived peptides in an exogenous context. Lastly, the model may involve isoform 3 influencing the recycling pathways of MHC class I molecules, further regulating their peptide-binding potential. This stepwise mechanism highlights isoform 3’s critical role in maintaining proper immune surveillance by ensuring MHC class I molecules present only peptides loaded under controlled intracellular conditions. This effect underscores the complexity of immune responses and their potential implications for autoimmune diseases and immunotherapies.

Thus, in the context of our findings on isoform 3, the interplay between EVs, trogocytosis, and surrogate antigen processing and bystander killing effects illustrates a sophisticated network of antigen presentation mechanisms. EVs can carry a range of proteins and RNA that might influence the function of DCs and other immune cells, potentially enhancing the efficacy of antigen presentation (75). This suggests that immune cells expressing isoform 3 may acquire antigens not only through direct mechanisms like trogocytosis but also by utilizing the cargo from EVs to broaden their immunogenic repertoire.

Recognizing the role of alternative splicing in genes encoding the MHC class I peptide-loading complex underscore the flexible and adaptive nature of antigen processing pathways, suggesting that the immune system can utilize multiple mechanisms to effectively present antigens for CTL activation and recognition. This adaptability is especially important in responding to a wide range of pathogens and tumor antigens, as both efficient dendritic cell MHC class I cross-presentation of exogenous antigens and conventional MHC class I endogenous antigen presentation are crucial for effective immune responses (5, 7, 8, 22). Equally critical is controlling unintended T cell activation or cytotoxicity to prevent bystander T cell killing, which can lead to tissue damage and contribute to autoimmune responses (9, 12, 76).

## Data Availability

The original contributions presented in the study are included in the article/**Supplementary material**, further inquiries can be directed to the corresponding author/s.
